# Effect of occlusal splint and therapeutic exercises on postural balance of patients with signs and symptoms of temporomandibular disorder

**DOI:** 10.1002/cre2.136

**Published:** 2019-02-12

**Authors:** Simone S. I. Oliveira, Claudio M. Pannuti, Klenise S. Paranhos, João P. C. Tanganeli, Dalva C. Laganá, Newton Sesma, Marcos Duarte, Maria Luíza M. A. Frigerio, Sang‐Chon Cho

**Affiliations:** ^1^ Division of Prosthodontics, Center of TMD and Orofacial Pain, School of Dentistry Federal Fluminense University Brazil; ^2^ Division of Periodontics, School of Dentistry University of São Paulo Brazil; ^3^ Department of Periodontology and Implant Dentistry NYU College of Dentistry New York; ^4^ Department of Biophotonics Universidade of Nove de Julho Brazil; ^5^ Division of Prosthodontics, School of Dentistry University of São Paulo Brazil; ^6^ Department of Biomedical Engineering ABC Federal University Brazil

**Keywords:** force platform, mandible, occlusal splints, postural balance, posture, temporomandibular joint disorders

## Abstract

The aim of this study was to investigate the effects of the use of an occlusal splint on postural balance considering the occlusal splint as a device for treating temporomandibular joint disorder. A randomized, controlled, prospective clinical trial was conducted. The research group consisted of 49 patients (36 as test group and 13 as control group) between 18 and 75 years old, both genders, diagnosed as temporomandibular disorder by Research Diagnostic Criteria/Temporomandibular Disorders questionnaire and magnetic resonance imaging of the temporomandibular joints. Test group was treated with orientations for physiotherapeutic exercises and occlusal splint, whereas control group received orientation for physiotherapeutic exercises only. Postural equilibrium was evaluated by means of a force plate. After 12 weeks, the groups were re‐evaluated. Patients from both groups presented a significant increase in antero‐posterior speed with eyes closed, test group (*P* < 0.001) and control group (*P* = 0.046). Only patients of the test group presented a significant increase in antero‐posterior speed with eyes opened (*P* = 0.023). We concluded that the use of occlusal splint affected the postural balance.

## 1 BACKGROUND

It has been hypothesized that imbalances in neck muscles and anterior muscle chains, which encompass masticatory, trapezius, and pectoral muscles, may compromise the body while in a standing position (Ohlendorf, Seebach, Hoerzer, Nigg, & Kopp, 2014). The masticatory cycles should be balanced, once unilateral mastication consists of a source of imbalance for the neck muscles and anterior muscle chains, which could compromise the postural balance of body while in a standing position (Ohlendorf et al., 2014).

Posture is understood as the position of the human body considering its demonstration in space. It involves muscular activation controlled by the central nervous system, which allows postural adjustments as a result of a complex mechanism system controlled by integrated, multisensory input (visual, vestibular, and somatosensory; Kandel, Schwartz, & Jessell, 1991; Rubira et al., 2010). Disturbances in these functions are frequently observed in patients who complain of dizziness, being such one of the most common symptoms either in otological or in neurological clinics (Cuccia & Caradonna, 2009). The most frequently used technique to study postural control consists of evaluating the oscillation of body during erect and resting posture by means of a force platform (Nota, Tecco, Ehsani, Padulo, & Baldini, 2017; Rubira et al., 2010). Studies that use the electromyographic activity of masseter and temporal muscles (Nicolakis et al., 2001; Visscher, Huddleston Slater, Lobbezoo, & Naeije, 2000) have shown that displacement of the most anterior position of head may occur in individuals with temporomandibular joint disorder (TMJD) due to changes in jaw's resting position (Nicolakis et al., 2001). Changes in the mandibular position may be noticed in the proprioceptive afferents, which affect the center of pressure (COP) of feet as well as gait stability (Cuccia & Caradonna, 2009). Studies identified a prevalence of TMJD signs in subjects with Parkinson's disease and a high frequency of occlusal asymmetry (Silva et al., 2016). Some studies evaluated the association of the stomatognathic system over corporal posture, by means of correlation between malocclusion and posture (Armijo‐Olivo et al., 2011; Mason et al., 2018; Perillo et al., 2011). Further, some studies have shown the occurrence of a greater prevalence of ocular‐convergence defects in adults with TMJD when compared with healthy ones. These adults presented limited maximum opening, myofascial pain, pain in the shoulder, and pain in the neck area (Monaco et al., 2004; Monaco, Streni, Marci, Sabetti, & Giannoni, 2003). However, all of these studies are cross‐sectional and limit their results to the association between TMJD and posture. So far, there is no evidence of existence of a relationship between TMJD and posture (Boutron, Moher, Altman, Schulz, & Ravaud, 2008; Manfredini, Castroflorio, Perinetti, & Guarda‐Nardini, 2012), as well as the relationship between TMJD treatment and posture. Thus, the aim of this study was to investigate the effects of the use of an occlusal splint on postural balance considering the occlusal splint as a method of treating TMJD.

## 2 MATERIAL AND METHODS

The manuscript was prepared according to the Consort Extension Checklist for Non‐Pharmacologic Treatments (Yap, Tan, Chua, & Tan, 2002). This research was approved by the ethics committee of FOUSP (protocol 200/10) and registered at ClinicalTrials.gov under identifier NCT2251015.

This was a single blinded randomized controlled clinical trial, with two parallel arms. The study subjects were observed in two moments: before (baseline) and after intervention (12 weeks).

The study was conducted at the Occlusion and TMJ Clinic and at the “Envelhecer Sorrindo” Program of the Prosthodontics Department of the University of São Paulo's School of Dentistry. Of the 70 subjects, 21 were allocated to the control group and 49 were allocated to the test group. As a result of noise from the force platform, balance data were available for 49 patients (13 from control group and 36 from test group). Patients were aged between 18 and 75 years old, and there were 10 male and 39 female.

In order to be eligible for the trial, the patients had to be dentate and to present temporomandibular disorders (TMDs). As inclusion were TMDs diagnosed by means of RDC/TMD questionnaire (Research Diagnostic Criteria/Temporomandibular Disorders) defined Axis I diagnoses as myofascial pain, myofascial pain with limited opening, disc displacement with reduction, disc displacement without reduction with limited opening, disc displacement without reduction without limited opening, and arthralgia. Magnetic resonance imaging (MRI) is used for diagnosis of temporomandibular joints. The RDC/TMD questionnaire was applied by a single trained, calibrated examiner (SSIO). An exclusion criterion was osteoarthritis and osteoartrosis, which was diagnosed with magnetic‐resonance image of one or more of the following features: erosion of cortical bone, sclerosis of condylus or articular eminence, flattening of articular surface, and presence of osteophytes. Other exclusion criteria were continuous use of medications that could affect equilibrium, visual impairment, neurological problems, labyrinthitis, TMJ surgery history, and pregnancy.

Patients were randomized into test and control groups. Simple randomization was conducted by drawing cards in which it was written “occlusal splint with demonstrations for physiotherapeutic exercises” (test group) or “only demonstration for physiotherapeutic exercises” (control group). Patients and personnel were not blind to the treatment that was performed. On the other hand, the outcome assessor (MD) was blind for the treatment that each patient received.

Test group received occlusion splint and demonstration for therapeutic exercises. Occlusal splint was performed under the occlusal stability criteria (simultaneous bilateral contacts with absence of interferences in canine and anterior guides). The occlusal splint was made of vacuum‐formed 1.5‐mm‐thick acetate obtained by autopolymerizable acrylic resin, which was placed on the acetate in order to position the mandible in centric relation and to create simultaneous bilateral contacts, canine guide, and anterior guide in the occlusal splint. The occlusal splint was used throughout night plus 4 hr during the day, being 2 hr in the morning and 2 hr in the afternoon. Occlusal splint was used for 12 weeks, only after it was adjusted and patients reported comfort.

The demonstration for therapeutic exercises sought to put the jaw in the resting position. Maxillary teeth should stay approximately 2 mm apart from mandibular teeth, whereas the tip of the tongue should be accommodated over the incisive papilla in the hard palate (without touching the teeth). Further, patients were instructed to perform 15 repetitions, three times a day, for 12 weeks, of repeated opening and closing movements, paying close attention to the position of the tongue during the exercises.

### 2.1 Procedures

The first part consisted of screening and filling out an anamnesis form, which contained questions related to overall health, use of medications, symptomatology of pain in the spinal column, muscular weakness, and postural imbalance as well as history of falls. The patients accepted were also required to present a diagnostic image via magnetic resonance imaging of TMJ. After that, patients filled out the RDC/TMD questionnaire.

The primary outcome of the study was postural balance during standing, with presence or absence of visual stimuli.

Sample size was based on a difference between groups of 0.5 as regards the primary outcome, with an estimated standard deviation of 0.7, power of 80%, and alpha of 5%. Considering those estimates, 25 subjects per group (total: 50 subjects) would be needed. Considering losses to follow‐up, we planned to include 70 subjects.

Postural balance was evaluated at Biophysics Laboratory of the School of Physical Education of University of São Paulo. Balance was assessed with the stabilometry test, by means of a force platform (OR6–WP, AMTI, USA), which consisted of two sensory situations: presence or absence of visual stimuli. For each situation, three attempts were performed randomly considering eyes opened (eo) or eyes closed (ec).

The individuals were oriented to keep their eyes fixed at a point located at a distance of 3.80 m ahead. Each sensory condition was measured for 45 s. The subjects were positioned over the platform, keeping their feet 10 cm apart (between one heel and the other) while the abduction between feet was 10°. The force platform simultaneously measures the three components of the force (Fx, Fy, and Fz) and three components of the moment (Mx, My, and Mz) in the three directions (x, y, and z: anterior–posterior (AP), medial–lateral, and vertical directions, respectively). These components allow the calculation of the COP, which is expressed as two coordinates in the force platform. These two coordinates are identified in relation to the demonstration of the subject in the AP and medial‐lateral directions.

The range of dislocation from the COP in the AP direction was evaluated in both conditions (eo and ec). This corresponds to the oscillation of the body during the 45‐s measurement, and it is presented in centimeters per second.

### 2.2 Statistical analysis

Categorical data were described by absolute (*n*) and relative (%) frequencies, and continuous data by statistics of mean, standard deviation (*SD*), median, minimum (min), and maximum (max) values. The comparisons of the measurements from the platform between the times were evaluated by paired *t* test, and the comparison between groups evaluated independent samples *t* test. In cases where normality was not met, nonparametric Wilcoxon test was used to compare experimental times, and the comparison between groups was evaluated by nonparametric Mann–Whitney test. The significance level of the tests was 0.05, and the software SPSS (Statistical Package for the Social Sciences) version 19.0 was used for all analysis.

## 3 RESULTS

The study started on July 2011, and the primary completion date was December 2011. The anthropometric characteristics of the 49 patients are presented in Table 1, and equilibrium clinical characteristics are shown in Table 2.

**Table 1:** Statistical description of the demographic characteristics

Gender; n, %	Male	10	20,4
	Female	39	79,6
Age (complete years); mean (*SD*), min–max		39.8 (16.3)	18–75
Weight; mean (*SD*), min–max		62.7 (12.5)	40–95
Height; mean (*SD*), min–max		1.62 (0.09)	1.47–1.78
Race/color; n, %	Asian or Pacific Islander	4	8,2
	Black/Mulatto	9	18,4
	White	33	67,3
	Indigenous	3	6,1
Level of education; n, %	Elementary school	5	10.2
	High school	15	30.6
	Higher education–1 year	29	59.2

**Table 2:** Statistical description of the equilibrium clinical characteristics

Difficulty in performing; n, %	9	18.4
Headache; n, %	33	67.3
Dizziness; n, %	15	30.6
Vertigo; n, %	8	16.3
Deficiency in hearing; n, %	3	6.1
Deficiency in sight; n, %	31	63.3

The analyses of dislocation from the COP are shown in Table 3. The patients of the test group presented a significant increase in antero‐posterior velocity from the COP with eyes open (*P* = 0.023) and eyes closed (*P* < 0.001). Control group did not present a significant increase in the eyes open (*P* = 0.249) primary outcome only with eyes closed (*P* < 0.046). There were no significant differences between groups after treatment. The analysis of the diagnosis of temporomandibular disorder by RDC/TMD Axis I in Table 4 showed significance in relation to the test group after the use of the occlusal splint and guidelines of therapeutic exercises. Table 5 shows the patients' answers regarding the RDC/TMD questionnaire.

**Table 3:** Analysis of the center of pressure displacement

		Group	Before	After	Paired *t* test *P* value
SDAP eo	Average (*SD*) Min–max	Control	1.97 (0.88) 0.77–3.25	1.99 (0.9) 0.94–3.6	0.701
	Average (*SD*) Min–max	Splint	1.83 (0.9) 1.26–2.15	2.02 (1.48) 1.11–2.43	0.551
	*t* test *P* value (between groups)		0.684	0.634	
SDAP ec	Average (*SD*) Min–max	Control	1.7 (0.46) 0.96–2.38	2.05 (0.89) 0.81–4.2	0.345
	Average (*SD*) Min–max	Splint	1.82 (0.94) 1.18–2.25	2.22 (1.58) 1.26–2.71	0.423
	*t* test *P* value (between groups)		0.856	0.526	
VAP eo	Average (*SD*) Min–max	Control	8.57 (5.44) 3.75–20.98	9.38 (4.46) 3.51–17.72	0.249
	Average (*SD*) Min–max	Splint	7.21 (4.51) 4.9–7.32	8.68 (3.6) 6.14–11.25	0.023*
	*t* test *P* value (between groups)		0.390	0.856	
VAP ec	Average (*SD*) Min–max	Control	8.42 (5.97) 4.07–25.38	9.18 (3.63) 4.69–18.43	0.046*
	Average (*SD*) Min–max	Splint	6.79 (3.71) 5.3–7.23	9.53 (3.88) 6.69–11.52	<0.001*
	*t* test *P* value (between groups)		0.603	0.786	

*Note*. Statistical description for the comparison between time. Data are presented as SDAP: measurements of the standard deviation; VAP: velocity of the center of pressure; eo: measurement of the balance with eyes open; ec: conditions of the measure of the balance with eyes closed.

* Significant at α = 5%.

**Table 4:** Analysis of the diagnosis of temporomandibular disorder by RDC/TMD Axis I

	Time			Group	
	Total	Control	Test	Time 1	Time 2
Myofascial pain	**<0.001**	**0.046**	**<0.001**	0.147	**<0.001**
Right disc displacement	0.445	0.257	**0.007**	0.539	**0.004**
Left disc displacement	0.403	0.564	**0.058**	0.296	0.423
Right arthralgia	**0.002**	1.000	**0.002**	0.992	**0.005**
Left arthralgia	**0.004**	0.317	**<0.001**	0.243	**0.025**

Significant variables are set in bold.

**Table 5:** Comparison between groups and experimental as regards measures of signs and symptoms of Axis I RDC/TMD

	Between time (W)			Between group (MN)	
	Total	Control	Splint	Time 1	Time 2
01—Do you have pain on the right side of your face, the left side or both sides?	**<0.001**	0.157	**<0.001**	0.353	**<0.001**
02d—Could you point to the areas where you feel pain?	**<0.001**	1.000	**<0.001**	0.381	**<0.001**
02e—Could you point to the areas where you feel pain? (left)	**<0.001**	0.705	**<0.001**	0.065	**0.012**
03—Opening pattern	**<0.001**	0.063	**<0.001**	0.568	**0.002**
04a—Unassisted opening without pain	**<0.001**	0.095	**<0.001**	0.764	**<0.001**
04b—Maximum unassisted opening	**<0.001**	0.082	**<0.001**	0.099	**0.036**
04ba—Muscles pain	**<0.001**	0.109	**0.002**	0.616	**0.001**
04bb—Jaw joint pain	**0.039**	0.066	**0.002**	0.155	**<0.001**
04c—Maximum assisted opening	**<0.001**	0.106	**<0.001**	0.266	**0.009**
04ca—Muscles pain	**<0.001**	0.317	**0.001**	0.986	**0.004**
04cb—Jaw Joint pain	**0.003**	0.655	**0.003**	0.141	0.269
04d—Vertical incisal overlap	0.317	0.317	1.000	0.155	0.136
05ad1—Opening (right)	**0.017**	0.655	**0.005**	0.476	**0.007**
05ae1—Opening (left)	**0.002**	1.000	**<0.001**	0.575	**<0.001**
05bd1—Closing (right)	0.110	0.276	**0.013**	0.566	**0.059**
05be1—Closing (left)	**0.008**	1.000	**0.003**	0.438	**0.105**
05cd—Reciprocal click eliminated on protrusive opening (right)	**<0.001**	0.564	**<0.001**	0.948	**0.003**
05ce—Estalido recíproco eliminado durante abertura (left)	**0.001**	1.000	**<0.001**	0.457	0.081
06a—Right lateral excursion	**<0.001**	0.196	**<0.001**	0.084	**0.039**
06a2—Right lateral excursion—muscles pain	**0.039**	1.000	**0.023**	0.238	0.115
06a3—Right lateral excursion—jaw joint pain	**0.015**	0.317	**0.023**	0.408	1.000
06b—Left lateral excursion	**<0.001**	**0.027**	**<0.001**	**0.006**	0.292
06b2—Left lateral excursion—muscles pain	**0.010**	0.317	**0.008**	0.853	**0.003**
06b3—Left lateral excursion—jaw joint pain	0.131	0.317	0.083	0.290	**0.096**
06c—Protrusion	**<0.001**	0.168	**<0.001**	0.313	**0.007**
06c2—Protrusion (muscles pain)	0.516	0.102	0.083	0.290	**0.003**
06c3—Protrusion (jaw joint pain)	0.165	0.317	**0.023**	0.125	0.096
06d—Midline deviation‐right	0.180	1.000	0.180	0.467	0.564
06d2—Midline deviation‐left	0.276	1.000	0.276	0.239	0.353
07da—Right sounds—excursion right	**0.041**	1.000	**0.041**	0.125	0.548
07db—Right sounds—excursion left	1.000	1.000	1.000	0.391	0.391
07 dc—Right sounds—protrusion	**0.014**	0.180	**<0.001**	0.589	**0.001**
07ea—Left sounds—excursion right	0.317	1.000	0.317	0.391	0.548
07eb—Left sounds—excursion left	**0.046**	1.000	**0.046**	0.125	0.391
07ec—Left sounds—protrusion	0.273	0.317	0.196	0.247	0.718
08ad—Temporalis (posterior) right	**0.005**	1.000	**0.004**	0.360	**0.018**
08ae—Temporalis (posterior) left	**0.004**	0.317	**0.004**	0.314	**0.018**
08bd—Temporalis (middle) right	**0.007**	0.317	**0.011**	0.954	**0.003**
08be—Temporalis (middle) left	**0.007**	0.317	**0.011**	0.972	**0.003**
08 cd—Temporalis (anterior) right	**0.004**	0.783	**0.003**	0.862	**<0.001**
08ce—Temporalis (anterior) left	**<0.001**	0.083	**0.003**	1.000	**0.016**
08dd—Masseter (superior) right	**<0.001**	0.180	**<0.001**	0.805	**<0.001**
08de—Masseter (superior) left	**<0.001**	0.414	**0.001**	0.843	**0.004**
08ed—Masseter (middle) right	**<0.001**	0.705	**<0.001**	0.679	**<0.001**
08ee—Masseter (middle) left	**<0.001**	0.453	**<0.001**	0.715	**<0.001**
08fd—Masseter (inferior) right	**0.003**	0.317	**0.004**	0.259	**0.028**
08fe—Masseter (inferior) left	**<0.001**	0.705	**<0.001**	0.230	**<0.001**
08gd—Posterior mandibular region (right)	**0.009**	0.593	**0.004**	0.229	**<0.001**
08ge—Posterior mandibular region (left)	**0.018**	1.000	**0.003**	0.985	**0.047**

*Note*. W: Wilcoxon test; MW: Mann–Whitney test.

Significant variables are set in bold.

## 4 DISCUSSION

In this study, we observed that occlusion splint and demonstration for therapeutic exercises resulted in a significant increase in antero‐posterior velocity from the COP, both with eyes open and closed. In other words, occlusion splint provided an additional effect in postural control.

Tracking the clinical and anthropometric characteristics of patients is important in studying TMJD and postural balance. Factors such as age, sex, weight, and height may influence both the efficiency of the treatment and the postural balance.

In this study, the sample was made up mostly of women (79.6%), which corroborated the literature in the prevalence of TMJD (Costen, 1997; Franco et al., 2010; Milani, De Periere, Lapeyre, & Pourreyron, 2000; Shetty, Pitti, Badu, Kumar, & Deepthi, 2010; Wahlund, 2003). Nevertheless, it is important to point out that there are controversies in relation to the uniform distribution between men and women (Hilgenberg, Saldanha, Cunha, Rubo, & Conti, 2012; Perillo et al., 2011).

There was no significant difference between groups as regards weight and height. Furthermore, there was no difference in relation to age, although a greater proportions of patients with TMJD occurred within the age range of 20 to 40 years (46.9%; Shetty et al., 2010). We believe that educational level (40.8% had at least 5 years of higher education) aided in more accurate perception and comprehension when responding the questionnaire.

A relevant characteristic of our study was the high prevalence of visual deficiency (63.3%), which corroborates other studies (Franco et al., 2010) that have shown the existence of a relationship between dental occlusion, the oculomotor system, and visual stabilization. The postural adjustments were observed as resulting from a complex system of mechanisms controlled by multisensory (visual, vestibular, and somatosensory) input integrated into the central nervous system (Rubira et al., 2010). Studies that relate ocular defects to TMJD (Monaco et al., 2004) corroborate our study. When comparing correlations between balance and visual deficiency, our study did not observe significant differences between group measurements comparing them with or without visual deficiency.

As a factor, age influenced the characteristics related to difficulty in performing rapid movements, which presented significant differences in patients 65 years old or older (8.2%) who presented TMJD (18.4%).

Auditory deficiency associated with TMJD has been studied by various authors (Costen, 1997; Milani et al., 2000). The low frequency (6.1 = %) presented in this study might be due to the fact that auditory deficiency has been evaluated only by questionnaires.

Similarly, the symptoms of vertigo (16.3%) and dizziness (30.6%), considered relevant (Akhter et al., 2013; Hilgenberg et al., 2012; Perillo et al., 2011) in postural balance in patients with TMJD, did not present themselves as statistically significant because clinical evaluation was not performed.

The signs and symptoms of TMJD have a strong bibliographic basis such that they may not be different from those found in our study. We observed, in a significant way, data in relation to pain in the face; the presence of joint noise; the habit of clenching or grinding the teeth; the sensation of tiredness in the mandibular joint upon waking, buzzing, headache, muscular, and joint pain; and limitation in mandibular movements such as opening and lateral excursions.

A study (Nota et al., 2017) showed a significant difference in body postural stability in between subjects with myogenous TMD and healthy controls, in increase in the amplitude of sway area and sway velocity postural parameters in maximum intercuspidation and rest positions, with eyes open. Already, the present study showed that the effect of use of occlusal splint and guidelines of therapeutic exercises in subjects with TMD, for 12 weeks, that were observed an increase in the velocity of the COP displacement in the antero‐posterior direction under the conditions with eyes open and closed without a corresponding increase in the amplitude of sway can be interpreted as an attempt at postural readjustment. The increase in velocity of the postural sway might be interpreted as an increase in the frequency of corrections performed by the subjects to maintain postural balance. This increase in the frequency of corrections might have been triggered by the realignment of the posture of the head and neck due to the use of the occlusal splint. A new question now is whether these results reflect a temporary change in the perception of body posture, suggesting that postural balance will continue to change (or even to return to its original state) or not. A study investigating the subjects using the occlusal splint and guidelines of therapeutic exercises for a longer time is needed to address this question.

A limitation of this study was the lack of sample size calculation and the simple randomized allocation, which produced groups with different sample sizes. On the other hand, this is the first study that investigated the effect of occlusal splint and guidelines of therapeutic exercises on postural balance. Still, more randomized trials are needed in order to prove this effect.

The effects of the use of the occlusal splint and guidelines of therapeutic exercises found in this work evince the importance of investigating TMJD and the consequences of treating it with a multidisciplinary approach.

## 5 CONCLUSIONS

There was an additional beneficial effect of the use of occlusal splint on the postural balance and guidelines of therapeutic exercises, with a significant increase in antero‐posterior velocity of COP of the body with eyes open and closed.

## CONFLICT OF INTEREST

The authors declare that there is no affiliation or any other conflict of interest. Implementation of the present study happened without any financial industrial support. There were no financial relationships between any of the authors and the manufacturers of products involved in the study. University of Sao Paulo, Brazil, and New York University School of Dentistry, United States of America, contributed equally to the research in this study and supported this study.

## CLINICAL TRIALS

**Figure 1:**
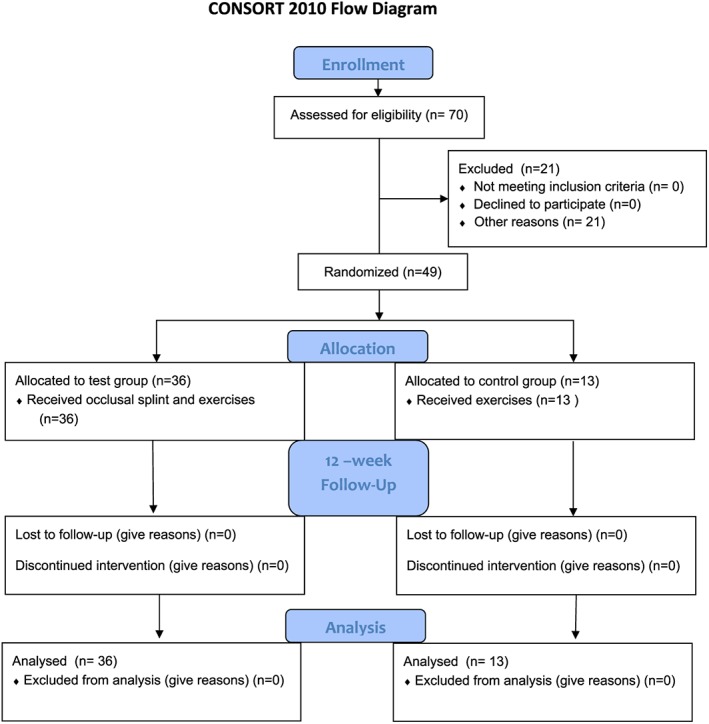
CONSORT flow diagram

This study is available at https://clinicaltrials.gov/, registered under ClinicalTrials.gov Identifier NCT2251015. CONSORT transparent reporting of trials by CONSORT flow diagram in Figure 1.
